# Treatment-seeking behaviour in low- and middle-income countries estimated using a Bayesian model

**DOI:** 10.1186/s12874-017-0346-0

**Published:** 2017-04-20

**Authors:** Victor A. Alegana, Jim Wright, Carla Pezzulo, Andrew J. Tatem, Peter M. Atkinson

**Affiliations:** 1 Geography and Environment, University of Southampton, Southampton, UK; 2 Flowminder Foundation, Stockholm, Sweden; 3 Faculty of Science and Technology, Lancaster University, Lancaster, UK; 4 School of Geography, Archaeology and Palaeoecology, Queen’s University Belfast, Belfast, BT7 1NN Northern Ireland UK

**Keywords:** Bayesian hierarchical model, Treatment-seeking behaviour, Item response theory, Markov Chain Monte Carlo

## Abstract

**Background:**

Seeking treatment in formal healthcare for uncomplicated infections is vital to combating disease in low- and middle-income countries (LMICs). Healthcare treatment-seeking behaviour varies within and between communities and is modified by socio-economic, demographic, and physical factors. As a result, it remains a challenge to quantify healthcare treatment-seeking behaviour using a metric that is comparable across communities. Here, we present an application for transforming individual categorical responses (actions related to fever) to a continuous probabilistic estimate of fever treatment for one country in Sub-Saharan Africa (SSA).

**Methods:**

Using nationally representative household survey data from the 2013 Demographic and Health Survey (DHS) in Namibia, individual-level responses (*n* = 1138) were linked to theoretical estimates of travel time to the nearest public or private health facility. Bayesian Item Response Theory (IRT) models were fitted via Markov Chain Monte Carlo (MCMC) simulation to estimate parameters related to fever treatment and estimate probability of treatment for children under five years. Different models were implemented to evaluate computational needs and the effect of including predictor variables such as rurality. The mean treatment rates were then estimated at regional level.

**Results:**

Modelling results suggested probability of fever treatment was highest in regions with relatively high incidence of malaria historically. The minimum predicted threshold probability of seeking treatment was 0.3 (model 1: 0.340; 95% CI 0.155–0.597), suggesting that even in populations at large distances from facilities, there was still a 30% chance of an individual seeking treatment for fever. The agreement between correctly predicted probability of treatment at individual level based on a subset of data (*n* = 247) was high (AUC = 0.978), with a sensitivity of 96.7% and a specificity of 75.3%.

**Conclusion:**

We have shown how individual responses in national surveys can be transformed to probabilistic measures comparable at population level. Our analysis of household survey data on fever suggested a 30% baseline threshold for fever treatment in Namibia. However, this threshold level is likely to vary by country or endemicity. Although our focus was on fever treatment, the methodology outlined can be extended to multiple health seeking behaviours captured in routine national survey data and to other infectious diseases.

## Background

Delay in seeking treatment for ill health in low- and middle-income countries (LMICs) affects disease progression, management and outcomes [1–3]. Most infectious diseases in LMICs are preventable by using cost-effective interventions and treatable at peripheral health facilities [4]. However, weak health systems affect the delivery of most interventions [5] and socio-economic and physical barriers that modify health-seeking behaviour compound this, leading to under-utilisation of health facilities [6]. Encouraging appropriate treatment-seeking behaviour for uncomplicated infections is vital to further reduce disease burden in these countries or for successful elimination. For malaria, for example, the current World Health Organisation (WHO) recommendation is for malaria treatment to be sought in the formal healthcare sector within 24 hours of fever onset and other malaria-related symptoms [7]. This is because patients who seek treatment through the formal sector are likely to receive an appropriate diagnosis and effective management [8]. However, there are many factors influencing population treatment-seeking behaviour including, but not limited to; availability of healthcare providers, proximity or travel time to healthcare facilities, condition severity and perception, and the socio-demographic profile of the population at risk [9].

Studies on treatment-seeking behaviour can be grouped into two categories of approach. The first is a qualitative description of steps undertaken by the population in different settings [10–12] while the second is a quantitative association between determinants (factors) and choice of health service use [13–18]. Although these approaches are used widely in bio-medical research, they usually do not examine the latent (i.e. theoretical) characteristics such as individual-level traits to estimate variation at population level. In addition, comparability is not simply guaranteed with the same questionnaire because of differential item functioning problem i.e. the varying behavioural response to the same question depending on the respondent [19]. Such variation can then be translated to spatially explicit applications that can be combined with existing spatial data on populations [20] and disease incidence to inform and optimise targeting of community-based interventions.

Model-based geostatistical methods have already been used to predict and estimate disease incidence at fine spatial resolution [21, 22]. This has been aided by public health intelligence data that are increasingly becoming available across space and time from geo-located nationally representative household surveys. These include the Malaria Indicator Surveys (MIS) [23], Demographic and Health Surveys (DHS) [24], and Multiple Indicator Cluster Surveys (MICS) [25]. These nationally representative household surveys also collect information on self-reported health behaviour such as fever management [14]. However, how can responses concerning fever treatment from household surveys be compared across populations with varying access, demographics, cultures, and disease burdens? Item response theory (IRT) has been widely used to examine surveys items (questions) and person characteristics in psychology and education [26–28]. In education, for example, it has been used to estimate the person-level traits (such as ability) or item-level difficulty in an examination [29–31]. IRT concepts can be extended to health as applied previously in delirium screening [32], longitudinal data analysis [33], and interpreting medical codes from patient records [34]. IRT approaches are essentially probit models with additional regression effects used to aid estimation of item characteristics [35]. Extending this to a Bayesian framework has the advantages of incorporating uncertainty in estimating latent traits and prior distributions can be imposed on the Bayesian probability model to capture many aspects of data not included in descriptive or quantitative frequentist approaches [36]. Although Imputation techniques can be used to handle missing data, this was beyond the current scope of this manuscript.

Here, the aim was to demonstrate the application of IRT to fever treatment-seeking modelling using data from a low malaria transmission setting, the Namibia 2013 DHS. We analyse fever treatment-seeking behaviour at a national level and derive response characteristic curves based on travel times to the nearest facilities. The rest of this paper is organised as follows. Section 2 provides an overview of household survey data in LMICs and the proposed modelling approach. We then present treatment-seeking behaviour model outputs in section 3, including evaluation of model performance. The paper concludes with a brief discussion in sections 4 and 5.

## Methods

### Data characteristics in low- and middle income countries

Distance or proximity to healthcare provider is an important parameter in the choice of treatment by patients in many LMICs [37–39]. In these countries, the majority of people access facilities by walking. Therefore, it is preferable to use a facility close to the place of residence because it is less costly compared to travelling greater distances requiring motorised transport [40]. Other factors that influence utilisation patterns include: age, gender, healthcare costs, socio-economic status, residence (urban or rural), familiarity with health personnel, fever severity, and quantity as well as quality of services at peripheral facilities [41, 42]. In some cases, however, the phenomenon of by-passing the nearest healthcare facility can be encountered, even for mild fever conditions [43, 44]. Empirical data are not always available to model such nuances and we therefore assume use of the nearest facility in this case study.

### Estimation of travel times to the nearest formal healthcare treatment provider

Estimating travel times between population centres and formal healthcare providers has already been considered in previous research [14]. In brief, this requires a combination of mode of travel (walking or motorised) and an impedance surface that is constructed based on multiple data layers, including the various land use and land cover characteristics, elevation, and roads [45]. Travel time to nearest healthcare facility is a useful measure because it is relatively easy to estimate and to relate travel times in different settings compared to estimating the actual physical distance. The approach in Alegana et al. [14] shows how travel times for Namibia were derived.

### Quantification of formal healthcare use based on national representative household surveys

To estimate the utilisation of healthcare facilities, this study used the reported use of formal healthcare for fever treatment from the DHS. These surveys are conducted in 90 countries worldwide, and 44 in SSA, providing information on reproductive health, fertility, population demographics and general health status, nutrition, household characteristics, socio-economic status and infant and child mortality rates [46]. The surveys are based on a random two-stage cluster sampling design in which clusters are usually first sampled within a region on a probability-proportional-to-size basis and thereafter, within each cluster, households are sampled randomly [47, 48]. Cluster sizes usually vary, but are typically approximately 15 to 30 households. The household survey provides information on health and the socio-demographic profile of consenting participants including their treatment-seeking behaviour for conditions such as malaria-associated fever.

**Fig. 1:**
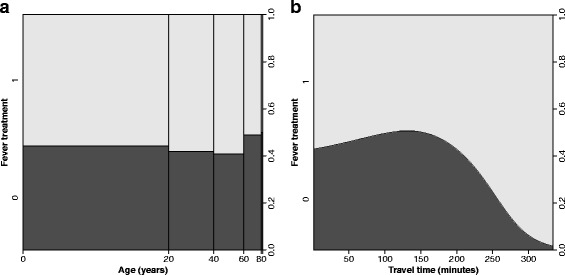
Visualisation of malaria-associated fever treatment from DHS data by **a** age (Children 0–5 years) and **b** by travel time to the nearest health facility generated from GIS methods combining spatial data (Land cover, roads), population centres and the locations of health facilities

A notable feature of the fever treatment variable in the DHS is the decay in treatment with increasing travel time to nearest facility (Fig. 1). The geographical barrier to utilisation, manifested as a distance decay, is a well-known phenomenon in studies of healthcare utilization [37, 38, 49] and occurs when usage of health facilities declines with increasing distance [50, 51]. This feature motivates the use of probit models to characterise treatment-seeking behaviour (section 2.4). Another feature of utilisation is that even for patients in close proximity to healthcare facilities, treatment for fever is not always 100% as some mild conditions self-resolve, are treated through informal care, or may be treated at a more distant facility [9]. Household survey data usually contain detailed information on other factors that could affect utilisation of healthcare facilities. These explanatory variables can be grouped largely into socio-economic and demographic characteristics and have been used selectively in quantitative studies of healthcare utilisation [3, 10, 17, 52, 53].

### Application of Bayesian probit models to healthcare utilisation research

Item response modelling was proposed in the 1960s [54–56] and is commonly applied to studies in education and psychology to estimate item characteristics [28]. The first applications of IRT used maximum likelihood estimation [57, 58]. Bayesian extensions were proposed for one- and two-parameter models [59] and extended to the three-parameter logistic model [60]. Fitting via Gibbs sampling became popular using data augmentation (DAG) techniques in the 1990s particularly for application to the normal-ogive models [61–63]. Fu et al. [64] provided some extensions to the three-parameter model following Sahu’s DAG approach [63] and compared Gibbs sampling to BILOG-MG software [65] using likelihood estimation. There have also been other innovations in parameter estimation [62], including extension to a multi-level approach [26–28] and comparison with maximum likelihood methods [66]. Here, a unidimensional three-parameter model with a hierarchical structure was used, its parameters estimated, and prior sensitivity checked by comparing model goodness-of-fit statistics. The main objective was to estimate the probability of a positive response to choice of treatment for persons with fever associated with malaria at a household level.

In general, let *Y*_*ij*_ represent a dichotomous response variable of an individual *j* (*j* = 1,......, *N*) on a set of questions (items) *i* (*i* = 1,......, *n*) on use of public healthcare for treatment. *Y*_*ij*_ = 1 represents a positive response on one item (e.g. public healthcare use), while *Y*_*ij*_ = 0 represents a negative response (e.g., non-public healthcare use). The probability of *Y*_*ij*_ = 1 can then be written following [64] as:

1 $$ P\left({Y}_{i j}=1\Big|{\theta}_j,{a}_i,{b}_i,{c}_i\right)={c}_i+\left(1-{c}_i\right)\frac{ \exp \left\{{\displaystyle \sum_{k=1}^m\left({a}_{i k}{\theta}_{j k}-{b}_{i k}\right)}\right\}}{\left[1+ \exp \left\{{\displaystyle \sum_{k=1}^m\left({a}_{i k}{\theta}_{j k}-{b}_{i k}\right)}\right\}\right]} $$

where, *θ*_*j*_ = *θ*_*j*1_......, *θ*_*jk*_,..... *θ*_*jm*_ with − ∞ < *θ*_*jk*_ > + ∞ for *k* = 1,..... *m* dimension represents the person traits (i.e. the ability parameter). *a*_*ik*_ represents item discrimination parameters between individuals separated by individual-level traits, and is positive (*a*_*ik*_ > 0). *b*_*ik*_(−∞ < *b*_*ik*_ < ∞) represents item difficulty (or location) parameters which for multiple items represents relationship between items and the underlying individual-traits (see Appendix for full glosary of symbols). Lastly, *c*_*i*_ (0 < *c*_*i*_ < 1) represents the threshold (i.e., minimum) probability for the item in question (fever treatment). This specification of threshold probability is important to this application because the estimated probability is never equal to one when *θ*_*j*_ is zero, due to several individual characteristics. Hence, probability of treatment is constrained to be greater than zero and less than one. In many applications in psychology and education, the ability parameter, for example, is modelled as a latent characteristic independent of survey observations [67, 68]. In this application, a predictor variable was introduced on the individual traits parameter in terms of travel-time to the nearest health facility. This parameterisation also enables the introduction of other variables such as residence (urban or rural), socio-economic status, or educational levels. Thus, Eq. 1 can be simplified to:

2 $$ {P}_{i j}={c}_i+\left(1-{c}_i\right){\varPsi}_{i j} $$

Where

$$ \begin{array}{l}{\varPsi}_{ij}= \exp \left\{{\displaystyle \sum_{k=1}^m\left({a}_{ik}{\theta}_{j k}-{b}_{ik}\right)}\right\}/\left[1+ \exp \left\{{\displaystyle \sum_{k=1}^m\left({a}_{ik}{\theta}_{j k}-{b}_{ik}\right)}\right\}\right]\\ {}{\theta}_j={\alpha}_j+{\beta}_{1 j}{X}_{1 j}+........+{\beta}_{Qj}{X}_{Qj}\end{array} $$

with *β*_*Qj*_ representing coefficients of dependent variables *X*_*Qj*_ exploring differences in ability.

### The likelihood and posterior specification

In general, let *f*(*θ*, *a*, *b*, *c*) denote a collection of unknown parameters, the posterior can be expressed as the product of the likelihood and prior distributions for unknown parameters given as:

3 $$ f\left(\theta, a, b, c\Big| y\right)\propto L\left( y\Big|\theta, a, b, c\right) f\left(\theta, a, b, c\right) $$

where *f*(*θ*, *a*, *b*, *c*) = *f*(*θ*)*f*(*a*)*f*(*b*)*f*(*c*) and the posterior density we wish to evaluate is

$$ \begin{array}{l} D\times L\left( y\Big|\theta, a, b, c\right)\times \left\{{\displaystyle \prod_{j=1}^N f\left(\theta \Big|{\mu}_{\theta},{\sigma}_{\theta}^2\right)}\right\}\times {\displaystyle \prod_{i=1}^n f\left({a}_i\Big|{\mu}_a,{\sigma}_a^2\right)}\\ {}\times I\left({a}_{i k}>0\right)\times {\displaystyle \prod_{i=1}^n f\left({b}_i\Big|{\mu}_b,{\sigma}_b^2\right)}\times {\displaystyle \prod_{i=1}^n f\left({c}_i\Big|{\kappa}_i,{\tau}_i\right)}\end{array} $$

where D is a proportionality constant and

$$ \begin{array}{l} L\left( y\Big|\theta, a, b, c\right)={\displaystyle \prod_{i=1}^n{\displaystyle \prod_{j=1}^N\left[{P}_{i j}^{y_{i j}}{\left(1-{P}_{i j}\right)}^{1-{y}_{i j}}\right]}}\\ {} and\\ {}{\displaystyle \prod_{j=1}^N f\left(\theta \Big|{\mu}_{\theta},{\sigma}_{\theta}^2\right)}={\displaystyle \prod_{j=1}^N \exp \left\{-\frac{1}{2}{\left({\theta}_j-\mu \right)}^T{\varSigma}_{\theta}^{-1}\left({\theta}_j-\mu \right)\right\}}\\ {}{\displaystyle \prod_{i=1}^n f\left({b}_i\Big|{\mu}_b,{\sigma}_b^2\right)}\times {\displaystyle \prod_{i=1}^n f\left({a}_i\Big|{\mu}_a,{\sigma}_a^2\right)}=\frac{1}{\sigma^2}{\displaystyle \prod_{i=1}^n}{\displaystyle \prod_{k=1}^m\frac{1}{\sigma^2}} \exp \left\{-\frac{{\left({a}_{i k}-{\delta}_a\right)}^2+{\left({b}_{i k}-{\delta}_b\right)}^2}{2{\sigma}^2}\right\}\\ {}{\displaystyle \prod_{i=1}^n f\left({c}_i\Big|{\kappa}_i,{\tau}_i\right)}={\displaystyle \prod_{i=1}^n{c}_i^{k-1}{\left(1-{c}_i\right)}^{\tau -1}}\end{array} $$

### Goodness-of-fit statistics, prior specification and Markov chain Monte Carlo implementation

The same notation was used for the item discrimination parameter, with*a*_*i*_ > 0, where a half-normal or truncated normal prior was used such that *a*_*i*_ ~ *N*(*μ*_*a*_, *σ*_*a*_^2^)*I*(*a*_*i*_ > 0)and *I*(⋅) is an indicator function. The rationale for this specification is to ensure that the parameter estimate is positive. The probability threshold parameter was constrained on *c* ∈ (0, 1] using a beta distribution such that *π*(*c*_*k*_; *κ*, *τ*)*αc*_*k*_^*κ* − 1^(1 − *c*_*k*_)^*τ* − 1^ for suitable parameters values ${\kappa}_{c_k}$and${\tau}_{c_k}$. The recommended procedure for selecting suitable estimates of these parameters is such that the *E*(*c*) = *κ*/(*κ* + *τ*) and weakly informative priors may be used for parameters of beta distribution.

**Fig. 2:**
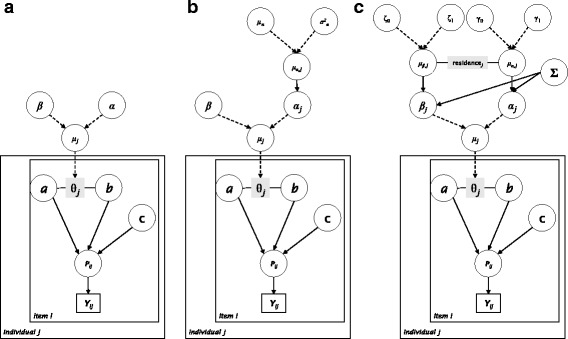
Graphical representation of the form of the models used. **a** simplified fixed parameter specification used for model 1 and model 2; **b** allowing for a random slope (model 3) on the *α*parameter; **c** random slope and intercept (model 4) for the *α* and *β* parameters, respectively, centering on residence (urban and rural) with correlation estimated via the Wishart prior specification. Model 1 and Model 2 differ only in the prior specification for item difficulty (*b*) parameter

Two different specifications were used for the difficulty parameter. The first was a normal prior *b*_*i*_ ~ *N*(0, 10) (model 1) and the second a truncated normal (model 2) restricting *b*_*i*_ ~ *N*(*μ*_*b*_, *σ*_*b*_^2^)*I*(*b*_*i*_ > 0) to be positive. Thus, the difference between model 1 and model 2 was only in the prior specification for the *b* parameter. Figure 2a represents the overall parameter structure for Model 1 and Model 2. The rationale for using different priors for *b* was to evaluate the effect of constraining item difficulty to positivity (*b*_*i*_ > 0) compared to allowing for flexible Gaussian density.

Lastly, the individual-trait parameter *θ*_*j*_ was modelled in a hierarchical approach following Fox and Glas [30] such that the joint distribution of *θ*_*j*_ parameters follows a multivariate normal distribution. Thus, in general, *α* and *β* are the intercept terms and regression coefficients, respectively, modelled as independent effects in model 1 and model 2 (Fig. 2a). In extending the model to a multi-level representation, time to the nearest facility could then be used to explain individual traits. Normal priors (e.g.*α* ~ *N*(0, 1)) were used for *α* and *β* in Fig. 2a. Secondly, this was extended to a random intercept in model 3 (Fig. 2b) and lastly, as a random slope and intercept model including residence (urban or rural) as a centering variable (model 4, Fig. 2c). For model 4, the random slope and intercept were modelled jointly as:

4 $$ \left(\begin{array}{c}\hfill {\alpha}_j\hfill \\ {}\hfill {\beta}_j\hfill \end{array}\right)\sim M V N\left\{\left(\begin{array}{c}\hfill {\beta}_{\alpha, j}\hfill \\ {}\hfill {\beta}_{\beta, j}\hfill \end{array}\right),\sum =\left(\begin{array}{cc}\hfill {\tau}_{11}\hfill & \hfill {\tau}_{12}\hfill \\ {}\hfill {\tau}_{21}\hfill & \hfill {\tau}_{22}\hfill \end{array}\right)\right\} $$

with a Wishart (multivariate scaled *χ* ^2^) distribution (Barnard et al. 2000) with density $f\left(\sum \right)\propto {\left|\sum \right|}^{-\left(\nu + d+1\right)/2 e-\frac{1}{2} tr\left(\wedge {\sum}^{-1}\right)}$; *d* dimension matrix; *ν*degrees of freedom; specified for covariance matrix ∑. Thus, the inverse is specified as ∑^− 1^ = *Wishart*(*Ω*, *p*) where *Ω* is a scale matrix, usually identity, and *p*is the degrees of freedom equal to the number of random components. Alternative approaches could employ a scaled inverse-Wishart distribution because of the large standard errors associated with large variances in the use of the inverse-Wishart prior [69].

Validation was considered via a subset of 40% of the data selected randomly (*n* = 247 of the 1138 children) with the remaining 60% (*n* = 891) used in model estimation. Model 1 was then applied to the validation set and the predicted probability of treatment transformed to a binary outcome. A receiver operating characteristic (ROC) curve was then used to derive the specificity and sensitivity of predictions when compared to observed responses from survey data. For estimation, different model specifications were also used to check the sensitivity of different prior specifications (i.e. models differ only on prior structure) and complexity. Model outputs were evaluated and compared via goodness-of-fit statistics, for example, the Deviance Information Criterion (DIC). The DIC summarises model fit based on a combination of model deviance and complexity (effective number of parameters) [70, 71]. This is defined as:

5 $$ D I C=\overline{D}+ p D $$

where $\overline{D}={E}_{\theta \Big| y}\left[ D\right]$is the mean deviance for *D* = − 2 × log{*P*(*y*|*θ*)} with

$$ \begin{array}{l}\overline{D}=-{\displaystyle \int 2 \log \left\{ P\left( y\Big|\theta \right)\right\}} d\theta \\ {}\widehat{D}=-2 \log \left\{ P\left( y\Big|\overline{\theta}\right)\right\}\end{array} $$

and complexity (effective number of parameters) given by $p D=\overline{D}-\widehat{D}$. The two parameters were monitored in the MCMC implementation using five chains in JAGS version 4.2.0 and the *rjags* package in R version 3.3.1 [72]. A combination of Gelman-Rubin [73] with Raftery-Lewis diagnostic [74] approaches were used to check for convergence. For the former, we checked for a reduction factor of <1.05 while the latter provided estimates of burn-in and thinning factors given an accuracy of 0.0005 at quantile (0.025) and coverage probability of 0.975.

## Results

We used the Namibia 2013 DHS data to estimate the probability of fever treatment in the formal sector (reported fever treatment in public and private sectors) for children under five years. There were 4818 children under five years enumerated, of which 1138 (23.6%) reported at least one fever episode in the preceding fortnight. Of those that reported a fever episode, 726 (63.8%) sought treatment in the formal sector (public and private sector excluding traditional healers). Overall, the proportion of children with reported fever was fairly homogeneous across all the regions surveyed but varied by estimated travel times. Estimation of probability of treatment focussed on children reporting fever (*n* = 1138) rather than all children examined in the cross-sectional survey.

In terms of computation, the Gelman-Rubin test was less than or equal to 1.05 for all the parameters monitored in the MCMC implementation. However, the Raftery-Lewis method showed that a minimum of 55,318 iterations were required to achieve an accuracy of 0.0005 at coverage probability of 0.999 with quantile at 0.05. More than 100,000 iterations with a burn-in of 50,000 were implemented. Table 1 shows the DIC estimates and the effective number of parameters from the four models implemented. Comparison between model 1 (M1 DIC 3615.9) and model 2 (M2 DIC 3685.1) suggests that using truncated normal priors for the *b* parameter did not improve model fit. Increasing DIC (for model 3 and 4) was also directly proportional to the increase in model complexity by including random intercept and slope. This also increased computational demands for M3 and M4 requiring at least 250,000 iterations with longer burn-in (slow convergence). The difference in DIC estimates also suggested that the models were sensitive to changes in model structure. Based on a binary classification of predicted probability at the individual level from model 1, the area under the curve (AUC) was 0.978 with a sensitivity of 96.7% and a specificity of 75.3% (155 true positive, 21 false positive, 64 true negative, and 7 false negative).

**Table 1:** Model comparison based on goodness-of-fit statistics

Model	DIC	PD	Inverse log likelihood	Number of chains
M1^a^	3615.9	2178.9	−0.001	3
M2^a^	3685.1	2256.1	−0.001	3
M3	5098.7	3693.1	−0.001	3
M4	23874.1	22754.0	−0.002	3

DIC is the deviance information criterion while PD is the model complexity (number of model parameters)

^a^Model 1 and model 2 only differ in prior specification for the *b* parameter

**Fig. 3:**
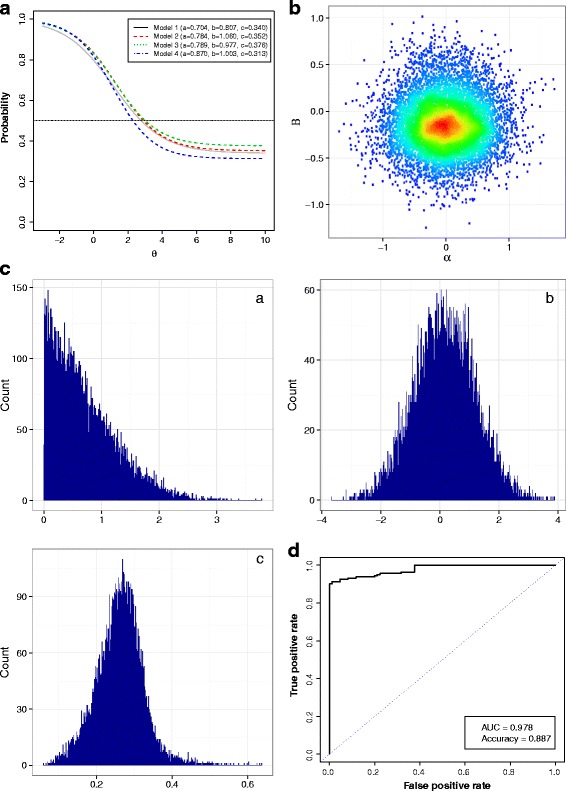
Panel plots showing. **a** Fever response decay curves from the four model parameter values from the DHS survey in Namibia for 2014. The data are from 1138 (*n* = 891 training, 247validation) children under the age of five reporting fever 2 weeks prior to survey of which 726 sought treatment in the formal sector. **b** A scatterplot for mean estimates on *α*(intercept) and *β* (slope) parameters based on model 4 (random slope and intercept model at individual level). **c** Posterior density for IRT parameters (*a*individual discriminant parameter, *b* item dificulty, and *c* probability threshold). **d** Receiver operating characteristic (ROC) plot based on the validation dataset (*n* = 247 children). The binary classification was based on the predicted probability of seeking treatment for fever (from model 1) with a cut-off at 0.65. ROC had AUC = 0.978 and an accuracy measure of 0.887

Table 2 shows posterior estimates of the parameters along with 95% equal-tailed credible intervals. A plot of fever-response curves based on the fitted parameters is shown in Fig. 3a along with a scatterplot of *α* and *β* parameters from Model 4 (Fig. 3b), posterior density of parameters (Fig. 3c) and ROC plot (Fig. 3d). Different mean combinations of parameters *a*, *b*, and *c* resulted in response characteristics based on travel time to nearest health facility (Fig. 3a). Parameter estimates could be compared and interpreted jointly in this manner because they apply to one item (on estimating fever treatment). Comparison between model 1 and model 2 suggested that constraining the *b* parameter did not have a major impact on mean estimates of the individual-level traits, *a* or the threshold parameter *c*. Overall, model 4 had larger person discriminant parameter estimates (mean and median) compared to all the other model specifications. The correlation between mean estimates for *α* and *β*as estimated from the model was weak (mean -0.011, median 0.006 scatterplot Fig. 2b). The combination of correlation and DIC estimates suggested a fixed prior independent specification as a better choice. It also imposes less computational demand. The threshold probability was >0.3 for all model estimates, suggesting this as the lower limit probability of use of nearest facility for fever treatment in the four models implemented from the 2013 Namibia DHS.

**Table 2:** Estimated summary statistics and the 95% Bayesian credible intervals of parameters based on all four models

Model	Estimate	a	b	c	α	β	Corr (α, β)
M1	Mean	0.704	0.807	0.340	−0.084	−0.098	-
	Median	0.556	0.850	0.326	−0.087	−0.112	-
	95% CI	[0.016–2.194]	[-1.044–2.346]	[0.1554–0.597]	[-0.682–0.523]	[-0.439–0.394]	-
	Gelman-Rubin Convergence estimate	1.000	1.000	1.000	1.000	1.000	-
	Gelman-Rubin Convergence upper CI	1.000	1.010	1.000	1.000	1.030	-
M2	mean	0.784	1.060	0.352	−0.080	−0.123	-
	median	0.654	0.978	0.344	−0.081	−0.121	-
	95% CI	[0.042–2.243]	[0.100–2.452]	[0.172–0.572]	[-0.661–0.518]	[-0.417–0.218]	-
	Gelman-Rubin Convergence estimate	1.001	1.000	1.001	1.001	1.020	-
	Gelman-Rubin Convergence upper CI	1.010	1.000	1.000	1.000	1.040	-
M3	mean	0.789	0.977	0.376	−0.582	−0.140	-
	median	0.660	0.895	0.372	−0.581	−0.150	-
	95% CI	[0.046–2.225]	[0.055–2.423]	[0.176–0.597]	[-2.208–1.069]	[-0.434–0.248]	-
	Gelman-Rubin Convergence estimate	1.000	1.000	1.000	1.000	1.000	-
	Gelman-Rubin Convergence upper CI	1.000	1.000	1.000	1.000	1.000	-
M4	mean	0.870	1.003	0.313	−0.133	−0.008	−0.011
	median	0.768	0.912	0.311	−0.152	−0.012	0.006
	95% CI	[0.059–2.244]	[0.063–2.477]	[0.095–0.527]	[-0.665–0.501]	[-0.880–0.873]	[-0.957–0.952]
	Gelman-Rubin Convergence estimate	1.000	1.000	1.000	1.010	1.000	1.010
	Gelman-Rubin Convergence upper CI	1.000	1.000	1.010	1.040	1.000	1.050

M1 and M2 use a fixed parameter specification for *α* and *β* using normal priors but different priors for item parameters, M3 allows random intercepts only, and M4 is both a random slope and intercepts model. Only M4 include a measure of correlation between the multi-level regression parameters

Table 3 shows the estimated mean probabilities for malaria related fever treatment at a regional level in Namibia with associated confidence intervals and population estimates. Population estimates are useful in estimating fever treatment burden based on probability estimate at regional level. For malaria, the probability of fever treatment among febrile cases was highest in endemic areas in Zambezi and Kavango (mean probability in Zambezi 0.546 (95% Credible Interval (CI): 0.369–0.671)) compared to Kunene with less than one case per 1000 population with mean probability 0.433 (95% CI: 0.364–0.614). Overall mean probability of fever treatment was greater than 0.5 in areas with malaria incidence >1 per 1000 population.

**Table 3:** Estimated probability for fever treatment (mean and 95% Bayesian Credible Interval) at the nearest health facility

Region	Population estimate 2015^a^	Estimated mean malaria incidence per 1000 population in 2014^b^	Estimated Average travel time to nearest health facility (minutes)	Probability of using a dispensary or clinic for fever treatment mean (95% CI)	Probability of using a health centre for fever treatment mean (95% CI)	Probability of using a Regional or district hospital for fever treatment mean (95% CI)
Zambezi	105,804	1.612	23.0	0.546 (0.369–0.671)	0.537 (0.369–0.667)	0.531 (0.369–0.661)
Kavango	259,984	1.467	29.7	0.513 (0.368–0.649)	0.498 (0.368–0.633)	0.503 (0.368–0.638)
Ohangwena	283,188	1.426	29.3	0.522 (0.368–0.650)	0.494 (0.367–0.630)	0.497 (0.368–0.632)
Oshikoto	210,881	1.256	37.3	0.504 (0.368–0.644)	0.492 (0.367–0.633)	0.496 (0.367–0.637)
Otjozondjupa	167,186	1.227	31.8	0.504 (0.368–0.643)	0.486 (0.367–0.623)	0.499 (0.368–0.637)
Omusati	281,050	1.131	35.6	0.513 (0.368–0.650)	0.497 (0.367–0.637)	0.498 (0.367–0.638)
Omaheke	82,441	1.126	38.3	0.490 (0.367–0.631)	0.496 (0.367–0.637)	0.493 (0.367–0.634)
Oshana	207,218	1.096	17.6	0.561 (0.369–0.677)	0.545 (0.369–0.661)	0.547 (0.369–0.663)
Kunene	102,986	0.967	146.4	0.433 (0.364–0.614)	0.426 (0.364–0.608)	0.429 (0.364–0.612)
Khomas^c^	418,742	-	43.9	0.487 (0.367–0.636)	0.483 (0.367–0.631)	0.482 (0.367–0.631)
Karas^c^	88,977	-	110.2	0.447 (0.365–0.619)	0.440 (0.365–0.613)	0.446 (0.365–0.618)
Hardap^c^	93,447	-	86.7	0.471 (0.366–0.628)	0.470 (0.366–0.626)	0.461 (0.366–0.616)
Erongo^c^	180,672	-	98.4	0.443 (0.365–0.620)	0.440 (0.365–0.618)	0.440 (0.365–0.617)

Data for health facilities represent public and private entities based on facility census 2009 [85] updated based on HMIS reports. Probability of use for fever treatment estimated from parameters of model 1

^a^Population estimates derived from worldpop [20]

^b^Mean malaria incidence derived from Alegana et al. [86]

^c^Regions designated as no malaria risk with case incidence of less than 1 per 10,000 population

## Discussion

Characterising treatment-seeking behaviour in LMICs is valuable because it varies by geographic location, type of disease and severity, person characteristics including age and gender, as well as health system based factors such as availability, cost among other enabling factors [9, 75, 76]. Here, the focus was on the estimation of latent parameters of a survey question on fever and estimating the probability of seeking treatment based on a dichotomous response. We used data from a nationally representative household survey from the DHS in one country to estimate fever treatment latent characteristics using a Bayesian IRT approach. By using this method, we estimated the parameters of fever response curves that characterise geographical decay in the use of formal health care based on travel time to the nearest facility. The method is particularly appealing because of the joint estimation of IRT parameters related to fever treatment with uncertainties incorporated in prior distributions and the ability to extract the full posterior distribution compared to point estimates from maximum likelihood approaches [26, 61]. This is important because estimates from such probabilistic modelling can then be applied in estimating numbers of symptomatic infections (treatment burden) when such probabilistic estimates are transformed into gridded metrics that vary spatially [77, 78]. The modelling approach can also be extended to other items in household surveys to further understand human behaviour response to health conditions.

The lower limit probability estimated here, related to the threshold parameter (e.g. from Table 2 model 1: 0.340; 95% CI 0.155–0.597), for Namibia suggests that even at large distances from health facilities, there was still a 30% chance of individuals seeking fever treatment. We suggest that this is an important property in treatment-seeking behaviour for individuals living far from health facilities in Namibia, although this threshold may be different by country or endemicity and was not explored further in this analysis. In this study, estimates of probability of fever treatment at the regional level showed that the mean probability was highest in regions with relatively high incidence of malaria historically (Table 3). Another operational application of the probability response characteristics curves, derived from the latent parameters in Fig. 3a, could be in identifying areas where community health workers could be deployed [79, 80]. This, however, requires definition of a cut-off probability (*y*-axis on Fig. 3a), currently not established for malaria transmission settings, to delineate areas with limited access. Constraining the *b* parameter (item parameter) did not influence estimates of the individual-level traits and the threshold parameters. This is primarily because only one item was used in this application resulting in similar parameter estimate for the location parameter.

In extending the model to a multilevel framework, travel times were used as predictors. Comparison between constant intercept and slope model parameters with a random parameter model showed that the former resulted in shorter MCMC runs and better model fit compared to the latter (i.e., the random slope and intercept), which experienced slow convergence as the number of effective parameters increased exponentially. We are not discouraging use of a more complex modelling approach while estimating IRT parameters, but this highlights the increasing computational demands and efficiency related to increased complexity.

MCMC techniques were used to estimate and jointly interpret IRT parameters. The three-parameter logistic model [60] was particularly useful compared to the two-parameter model [59], because, the third parameter *c* represents the threshold probability on the fever response curve, ensuring that probability is always greater than or equal to zero. Despite the known benefits of IRT in other fields [28], this approach has seldom been applied to modelling human behavioural aspects for treatment-seeking behaviour. The current study was confined to patients’ responses to a fever question in household survey data and how latent (rather than observed) properties can be quantified in relation to patient behaviour and travel time. Dichotomous responses are common in many health surveys in LMICs and methods used here can be extended to other health conditions. Although we did not have to deal with missing data (NAs), several data imputation techniques can be used for non-ignorable NAs [81]. These may arise when there is lack of response, or, associated with refusal to participate or simply unobserved variable for survey items. When NAs are imputed into the data matrix, for example, these do not usually contribute to likelihood estimation [82] of the ability parameter and the higher the number of missing values the more likely that there will be an increase in uncertainty for the parameter estimate.

There exist some additional limitations aside from those related to computational speed and efficiency. While fever in the Namibia 2013 DHS was associated with malaria treatment, the survey data did not include a laboratory confirmation of malaria infection [83]. Moreover, the sampling methodology for children with fever in the DHS may be inferior because the survey is not powered for fever detection [47]. Most current surveys however incorporate rapid diagnostic tests (RDTs) and future identification of febrile cases could include laboratory results as a preprocessing step in identifying malaria-related fever cases. In addition, although prior specifications introduce a measure of uncertainty in a hierarchical way, assumptions in generating input data such as use of the nearest facility may not be sufficient in understanding treatment-seeking behaviour. It has been shown in separate population surveys that patients may bypass the nearest health centre due to various individual- or supply-based factors such as quality [84]. While an obvious recommendation is to include such effects, increasing model complexity to capture such differences may have an impact on computational efficiency as seen in model 3 and model 4. More importantly, identifying measures of quality of care in public or private health sectors can be challenging [40].

## Conclusion

In the context of fever treatment, we have demonstrated that there is potential to use nationally representative household data to provide a probabilistic measure of treatment using a Bayesian method. Our estimates of threshold probability apply to one low malaria transmission country and may be different in other countries with varying malaria endemicity. Future studies will aim to conduct such comparative analysis between and within countries via spatially varying parameters. The methodology can be extended to multiple human behavioural questions (items) related to health and demographics in the routine national survey data.

## Appendix

### Parameter notations

*j* Individual/person

*i* Item/survey question

*k* Dimension for items

*q* Dimension for dependent variables

*a* Discrimination parameter

*b* Difficulty parameter on items

*c* Probability threshold parameter

*θ* Individual trait/ability parameter

*P*(*Y*) Probability that event Y occurs

*I*(⋅) Indicator function for event in sample space

*E*(*X*) Expectation for random parameter *X*

*μ* Mean

*DIC* Deviance Information Criterion

$$ \overline{D} $$ Mean deviance

[{(⋅)}] Order of brackets

## Abbreviations

AUC: Area under curve
CHW: Community health worker
DAG: Data augmentation
DHS: Demographic health surveys
DIC: Deviance information criterion
iCCM: Integrated community-case management
IRT: Item response theory
LMICs: Low- and middle-income countries
MCMC: Markov chain Monte Carlo
MICS: Multiple indicator cluster surveys
MIS: Malaria indicator surveys
ROC: Receiver-operating characteristics
SSA: Sub-Saharan Africa
WHO: World Health Organization
